# Surface Acoustic Wave Sensor for C-Reactive Protein Detection

**DOI:** 10.3390/s20226640

**Published:** 2020-11-19

**Authors:** Ming-Jer Jeng, Mukta Sharma, Ying-Chang Li, Yi-Chen Lu, Chia-Yu Yu, Chia-Lung Tsai, Shiang-Fu Huang, Liann-Be Chang, Chao-Sung Lai

**Affiliations:** 1 Department of Electronic Engineering, Chang Gung University, Taoyuan 333, Taiwan; mjjeng@mail.cgu.edu.tw (M.-J.J.); mukta.shrm@gmail.com (M.S.); pterolulu@gmail.com (Y.-C.L.); a093812678536@gmail.com (C.-Y.Y.); liann@mail.cgu.edu.tw (L.-B.C.); cslai@mail.cgu.edu.tw (C.-S.L.); 2 Department of Otolaryngology-Head and Neck Surgery, Chang Gung Memorial Hospital, Linkou 244, Taiwan; shiangfu.huang@gmail.com; 3 Green Technology Research Center, Chang Gung University, Taoyuan 333, Taiwan; davenlee15@gmail.com; 4 Department of Public Health, Chang Gung University, Taoyuan 333, Taiwan

**Keywords:** surface acoustic wave, C-reactive protein, piezoelectric lithium niobate

## Abstract

A surface acoustic wave (SAW) sensor was investigated for its application in C-reactive protein (CRP) detection. Piezoelectric lithium niobate (LiNbO_3_) substrates were used to study their frequency response characteristics in a SAW sensor with a CRP sensing area. After the fabrication of the SAW sensor, the immobilization process was performed for CRP/anti-CRP interaction. The CRP/anti-CRP interaction can be detected as mass variations in the sensing area. These mass variations may produce changes in the amplitude of sensor response. It was clearly observed that a CRP concentration of 0.1 μg/mL can be detected in the proposed SAW sensor. A good fitting linear relationship between the detected insertion loss (amplitude) and the concentrations of CRP from 0.1 μg/mL to 1 mg/mL was obtained. The detected shifts in the amplitude of insertion loss in SAW sensors for different CRP concentrations may be useful in the diagnosis of risk of cardiovascular diseases.

## 1. Introduction

C-reactive protein (CRP) is routinely used as a biomarker to detect inflammation and to assess the future risk of cardiovascular diseases [1,2]. C-reactive protein is produced in the liver and binds to phosphocholine in damaged or pathogenic cell membranes, inducing an innate immune system response [3]. C-reactive protein concentration in normal human serum is less than 1 μg/mL, but it increases by hundreds of times due to infection and other acute conditions. However, different CRP concentrations generate different response variations. It has been used as a blood test to assess the health of the human body and shows significantly increased serum CRP levels at the site of inflammation [4]. Autoimmune infections, autoimmune diseases, or cancer could be responsible for this. In clinical practice, commonly used CRP detection techniques are radial immunodiffusion (RID), radioimmunoassay (RIA), immune nephelometry (IN), immune turbidimetry (IT), immunofluorescence, chemiluminescence immunoassay, and standard enzyme immunoassays such as enzyme-linked immunosorbent assays (ELISA) [5,6]. A fluorescence-linked immunosorbent assay (FLISA) approach was developed after ELISA to reduce the analysis time, because FLISA does not require the chromogenic reaction steps of ELISA [7,8]. Although these methods are well established with good reproducibility and sufficient sensitivity, they are time-consuming and expensive, which are seen as major limitations. To overcome these limitations, various biosensors for CRP detection analysis based on electrochemical and optical methods [9,10,11], surface plasmon resonance (SPR) [12,13,14], piezoelectric microcantilevers [15], and quartz crystal microbalance technology [16,17] have been reported. Salvo et al. [18] reported different types of sensors and biosensors for monitoring the concentration of CRP for cardiovascular applications, and some of these biosensors were also suitable for monitoring the wound status. These CRP biosensors were classified as field effect transistors; optical immune-sensors based on surface plasmon resonance; electrochemical sensors based on potentiometry, amperometry, and electrochemical impedance; and piezoresistive sensors, such as quartz crystal microbalances and microcantilevers. According to this report, CRP concentration was less than 8 μg/mL for the normal clinical condition, while higher values (with average CRP level ranging from 20 to 95.6 μg/mL) were used to distinguish between different critical conditions. However, only a few sensors had a wide range (up 35 to 100 μg/mL or 200 μg/mL) that allowed the full spectrum of wound status to be covered. In addition, Khan et al. [19] reported that many biomarkers (e.g., creatinine, CRP, interleukin-6, B-type natriuretic peptide, N-terminal pro B-type natriuretic peptide) may be used as forecasters of cardiac patients’ status post cardiac surgery. However, large variations have been observed within these biomarkers. Aviles et al. [20] reported that a high level of CRP is not only associated with the presence of atrial fibrillation but may also predict patients at increased risk for future development of atrial fibrillation. The median CRP value of 1.92 μg/mL (interquartile range of 0.97–3.41) was measured based on 897 developed postoperative atrial fibrillation patients. The patients with a higher value of CRP had higher adverse event risk. In this paper, we report the application of a surface acoustic wave (SAW) sensor for CRP detection of up to 1 mg/mL concentration.

In recent years, SAW components have been widely used in filters, resonators, mobile phones, television, satellite communications, electronic actuators, sensors, chemical analysis, etc. [21,22,23,24,25,26]. Acoustic wave probes based on horizontally polarized shear waves (HPSWs) have been found to be more suitable than vertically polarized shear waves for sensing application in liquids [27]. Love-wave immune-sensors were successfully used for bio-sensing on AT-cut quartz substrates supported by HPSWs [28]. Surface acoustic wave design has several inherent advantages, such as high sensitivity and portability, high analogy, mass producibility, and the ability to produce different frequency responses (10 MHz to 1 GHz) by changing the geometry of interdigital transducers (IDT) [29,30]. All of these advantages have attracted many researchers to study this field. The first SAW interdigital transducers on a piezoelectric substrate/film were proposed with the use of cascaded equivalent circuits [31]. Afterwards, several other techniques were developed that characterized SAW properties for the thin metal layer and the effect of metallic grating finger thickness [32,33]. When an alternating current (AC) signal is applied to the input IDT, which is transmitted through the surface of the piezoelectric material, the surface waves travel across the sensing area and are very sensitive to variations on the sensor surface. Afterwards, the wave signal is converted back into an electrical signal at another output IDT. The frequency or amplitude changes at the resulting output signal can be correlated to the corresponding mass and mechanical variations on the sensing surface.

In this paper, we fabricated a SAW sensor for CRP detection by using LiNbO_3_ material with piezoelectric properties. In the future, such sensors can be used for CRP detection in medical science to explore the surface acoustic wave components with high sensitivity and to establish an assessment of the risk of developing cancer and other chronic diseases. After fabricating the SAW structure, the bio-sensing area of the gold surface was immobilized by surface modification. We can measure the sensor response with different concentrations of CRP that show the different drift values (insertion loss). In this CRP detection technique, the electrical detection of various CRP concentrations from 0.1 μg/mL to 1 mg/mL was measured in a linear relationship.

## 2. Materials and Methods

### 2.1. Design and Fabrication

**Figure 1:**
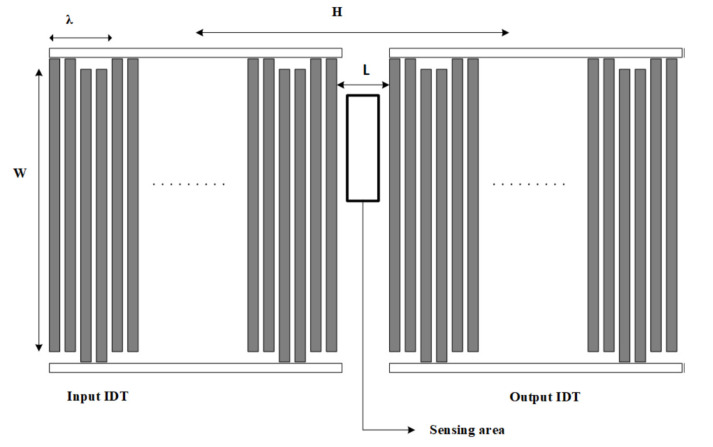
Surface acoustic wave (SAW) interdigital transducers (IDT) pattern.

**Figure 2:**
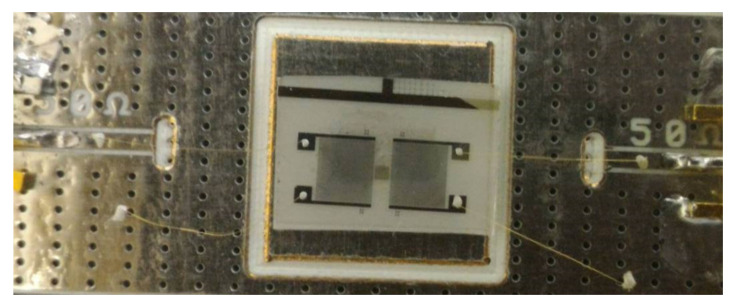
Fabricated SAW device connected to printed circuit board (PCB).

A SAW sensor was designed by two interdigital transducers—one for input and the other for output, as shown in Figure 1. The distance, L, between these two finger electrodes, was 1000 µm. Each IDT consisted of 100 equal-interval double-finger electrodes. The finger width of the IDTs was 8 µm with 8 µm separation, yielding 64 µm acoustic wavelength or periodicity (λ). The IDT aperture (W) was 3500 µm and the center-to-center distance, H, of the IDTs was 7392 µm. The bio-sensing area of SAW sensor consisted of 503 × 2628 µm dimensions. This IDT pattern was fabricated on a single 500-µm thick, single-side polished 128-degree Y-cut LiNbO_3_ (2 × 2 cm dimension). The aluminum (Al) electrodes with a thickness of 70 nm were evaporated using thermal evaporation. The patterning of the electrodes for the SAW IDT was performed by a conventional lift-off process. A guiding layer of silicon dioxide (SiO_2_) with 100 nm thickness was deposited on top of the device using electron beam evaporation for the protection of the IDTs or the isolation of the pattern. Finally, the bio-sensing area between two IDTs was deposited with a 10-nm thin layer of gold and 1 nm of chromium (Cr) adhesion layer by thermal evaporation and lift-off. A printed circuit board (PCB) was designed with 4.2 × 1.7 cm (chip area 1.2 × 1.2 cm) dimensions and the SAW device was connected to the PCB using wire bonding with silver glue, as shown in Figure 2.

### 2.2. Chemicals and Materials

Immobilization buffer (10 mM sodium acetate, pH 5.0), ProteOnTM amine coupling kit including 1-ethyl-3-(3-dimethylaminopropyl)-carbodiimide hydrochloride (EDC) and *N*-hydroxysuccinimide (NHS), and 1.0 M ethanolamine HCl (pH 8.5) were purchased from Bio-Rad (Piscataway, NJ, USA). Phosphate buffered saline (PBS) and 11-mercaptoundencanoic acid (11-MUA) were purchased from Sigma-Aldrich (St. Louis, MO, USA). Anti-C-reactive protein antibody (rabbit polyclonal antibody) and C-reactive protein antigen (Human, Recombinant) were purchased from Sino Biological.

### 2.3. Method of Immobilization

**Figure 3:**
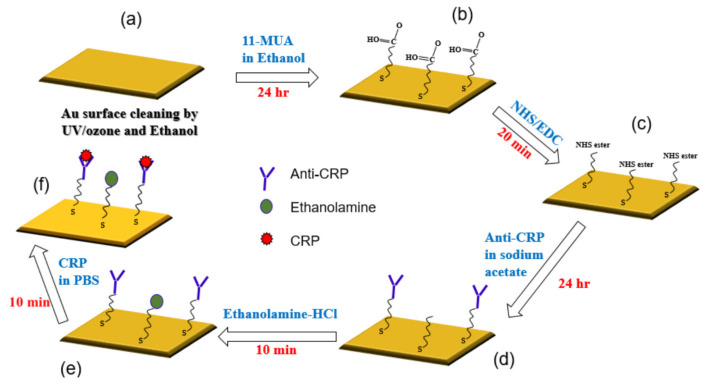
Schematic representation of C-reactive protein (CRP) and anti-CRP interaction process: (**a**) Au surface cleaning; (**b**) Self-assembled monolayer (SAM) on Au surface; (**c**) Activation of SAM; (**d**) Immobilization of anti-CRP; (**e**) Blocking the remaining non-specific binding of antibody active sites; (**f**) Evaluating the interaction between CRP and anti-CRP.

Figure 3 shows all the steps that were used for the immobilization of gold surface and the CRP/anti-CRP interaction process.

(a) The gold surface was cleaned by UV/ozone and ethanol and blown dry with nitrogen gas before use.
(b) For the surface modification binding, 11-mercaptoundencanoic acid (11-MUA) in absolute ethanol (4 mM) was injected into the gold sensing area and incubated for 24 h, then washed by absolute ethanol and deionized (DI) water, and dried by nitrogen (N_2_) gas. This was the way the self-assembled monolayer (SAM) was attached to the gold surface.
(c) This reactive SAM layer was activated by 1-ethyl-3-(3-dimethylaminopropyl)-carbodiimide (EDC)/N-hydroxy succinimide (NHS) mixture (1:1) for 20 min and washed by sodium acetate buffer (10 mM, pH 5.0).
(d) Immobilization of anti-CRP was done in sodium acetate buffer (10 mM, pH 5.0) for 24 h.
(e) Blocking was performed by incubating in ethanolamine-HCl (pH 8.5) solutions for 10 min and then washed and incubated in phosphate buffered saline (PBS) buffer. This process was done for blocking the remaining non-specific binding of antibody active sites or removing the interference.
(f) For evaluating the interaction between CRP and anti-CRP, the prepared CRP with PBS buffer solution was injected over the sensing area and allowed to react with anti-CRP for 10 min. We prepared the CRP solution of 10 mL individually for each CRP concentration (0.1, 1, 10, 100, and 1000 ug/mL), which was diluted by PBS buffer. It means that we had five bottles of CRP solution with different concentrations. The proposed SAW sensor was exposed to CRP solutions of increasing concentrations (0.1, 1, 10, 100, and 1000 μg/mL). We used a pipette to suck 50 uL from the prepared CRP solutions and cover its sensing surface to interact with the CRP antibodies for each measurement. After an interaction time of 10 min, the CRP solution was carefully removed by a pipette and the surface was rinsed with PBS (500 μL) and DI water (1000 μL), separately, for the removal of non-specifically bound molecules. Next, the residue water at the surface of the SAW sensor was also carefully removed by a low nitrogen gas flow for 30 sec. After the nitrogen drying process, the frequency response of SAW sensor for detecting the CRP was recorded by a vector network analyzer (VNA). We repeated three times the abovementioned four steps (CRP interaction, removal of CRP solution, surface drying, and record spectrum response) for each CRP concentration. It should be noted that surface liquid residues should be completely removed to avoid the radiation of the acoustic energy into the liquid. For Rayleigh SAW sensors, this will cause a severe attenuation of the acoustic wave propagating at the solid/liquid interface [34]. No resonance peak was observed when the surface liquid residues had not been removed.

**Figure 4:**
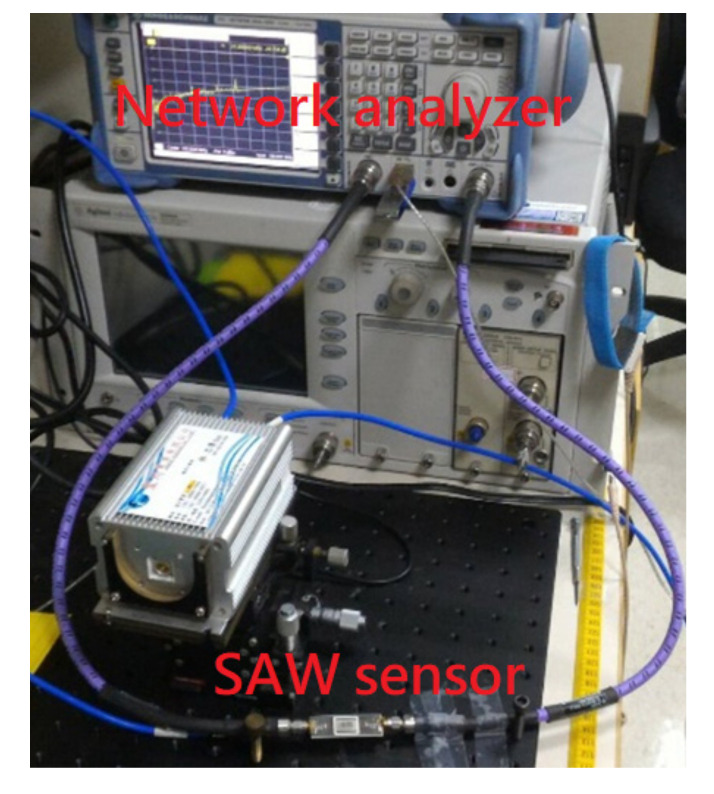
The measurement system for the frequency response of SAW sensors.

Figure 4 shows the measurement system for the frequency response of SAW sensors. A VNA was used to measure the frequency response of the SAW sensor. The calibration was done by a calibration kit before each measurement. All the measurements were done at room temperature. Port 1 of the VNA outputs high-frequency AC signals to the input terminal (the first SAW interdigital transducers) of the SAW sensor. The electrical signal is converted to surface acoustic wave by the first SAW interdigital transducer. The surface acoustic waves travel across the SAW sensing area and are very sensitive to variations on the SAW sensing surface. The surface acoustic wave is converted back to an electrical signal by the second SAW interdigital transducer. Port 2 of the VNA receives the signal from the output terminal (the second SAW interdigital transducer) of the SAW sensor. The frequency or amplitude response changes at the resulting output signal, which can be correlated to the corresponding mass and mechanical variations on the sensing surface.

## 3. Results and Discussion

**Figure 5:**
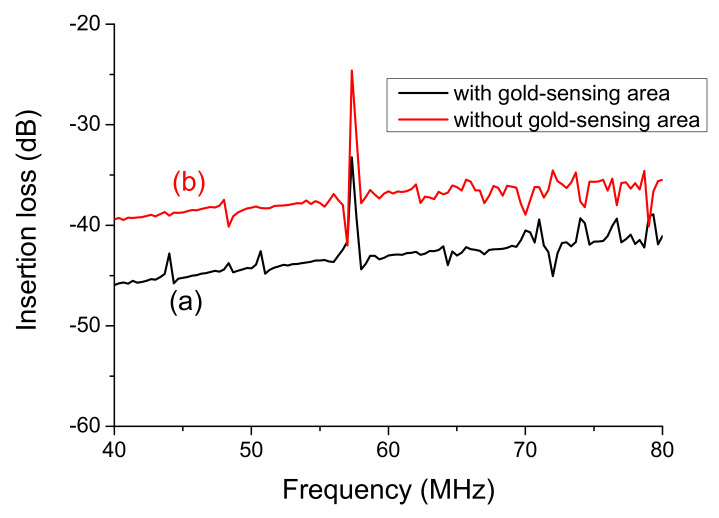
The frequency response of SAW sensors (**a**) with and (**b**) without the deposition of gold-sensing area on top of the SiO_2_ guiding layer.

Figure 5 plots the frequency response of SAW sensors with and without the deposition of the gold-sensing area on top of the SiO_2_ guiding layer. The fabricated 100 double pairs IDT SAW structure generated the center frequency at 57.34 MHz. The insertion loss of the SAW sensor without the deposition of the gold-sensing area was -26 dB, as shown in Figure 5b. After depositing the gold-sensing area on the SAW sensor, the insertion loss of the SAW sensor was observed as -33 dB, which is a reduction of 7 dB, as shown in Figure 5a. It is known that traveling surface acoustic waves are very sensitive to variations on the sensor surface. The deposition of the gold-sensing area may cause a mass-loading effect on the SAW sensor. Thus, the insertion loss attenuated to -33 dB, possibly due to the mass-loading effect caused by the deposition of the gold-sensing area. There are several very commonly used guiding layers, such as polymethylmethacrylate (PMMA), polydimethylsiloxane (PDMS), SiO_2_, and zinc oxide (ZnO) [35,36,37]. The guide layer can isolate and passivate the SAW devices. Silicon dioxide is the most promising material as a guiding layer, because it offers low damping, sufficient low shear velocity, and excellent chemical and mechanical resistance [38]. We selected SiO_2_ as a guiding layer for the confinement of acoustic energy and passivation of IDTs. Using this guiding layer, the acoustic energy of the sensor is confined in the guiding layer, keeping the wave energy trapped tightly near the surface, which makes the SAW sensor very sensitive to its sensing area [39].

After the fabrication of the SAW sensor, the immobilization process was performed for CRP/anti-CRP interaction. The CRP/anti-CRP interaction can be detected as mass variations on the sensing area. These mass variations may produce changes in the amplitude or phase of the sensor response. Different concentrations (0.1 μg/mL to 1 mg/mL) of standard CRP were tested to observe the frequency response of the SAW sensor as listed in Table 1. Measurements for each concentration were performed three times. The SAW sensor was cleaned by a large amount of PBS before every measurement. Interestingly, only the amplitude variation was observed with no apparent frequency shift.

Generally, the mass-loading effect in SAW sensors may change the amplitude and the center frequency. Because the thickness of the SiO_2_ guiding layer is much smaller than the SAW wavelength [40], it can be thought as an acoustically thin, elastic film in which the SAW-induced displacement is uniform across the film and varies only in the direction of propagation. The apparent change in insertion loss with no frequency shift in the proposed SAW sensor under different CRP concentrations is unclear now. However, a very small frequency shift was observed at a very high CRP concentration (1 mg/mL). There are several possible reasons for the lack of frequency shift. Martin et al. [40] reported that the mass response was only a small fraction of the total velocity shift response for a polymer-coated SAW device. In addition, Agostini et al. [34] reported that the resonance frequency deviates from its stable value if the surface liquid residues are not fully removed. The final possible reason is that the frequency shift is too small to be detectable in our measurement, because a very small frequency shift was observed at a very high CRP concentration (1 mg/mL).

**Figure 6:**
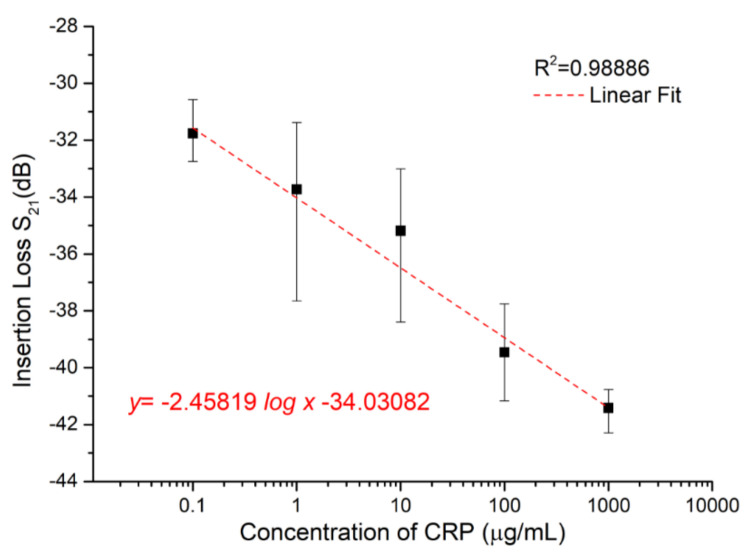
The correlation of amplitude attenuation and CRP concentrations of SAW sensors.

Figure 6 demonstrates the correlation of amplitude attenuation and CRP concentrations of SAW sensors. It was clearly observed that the CRP concentration of 0.1 μg/mL can be detected in the proposed SAW sensor. Aviles et al. reported that the median CRP value of 1.92 μg/mL (interquartile range 0.97–3.41) was measured [20]. In the proposed SAW sensor, the detectable CRP concentration of 0.1 μg/mL is high enough for clinical diagnosis because the clinically relevant range for detecting CRP concentrations is generally from 1 to 200 μg/mL [18]. The CRP sensitivity of a proposed SAW sensor should at least be 0.1 μg/mL based on the three trials for each concentration. A good linear relationship between the detected insertion loss (amplitude) and the concentrations of CRP from 0.1 μg/mL to 1 mg/mL was observed. The dark points in Figure 6 show the mean value of different amplitude (dB) for each CRP concentration. It was observed that the amplitude decreases with increasing CRP concentration due to the mass-loading effect. The mean value for each concentration can be fitted by a linear regression line. This regression line is useful to estimate the CRP concentration by using the proposed SAW sensor.

**Figure 7:**
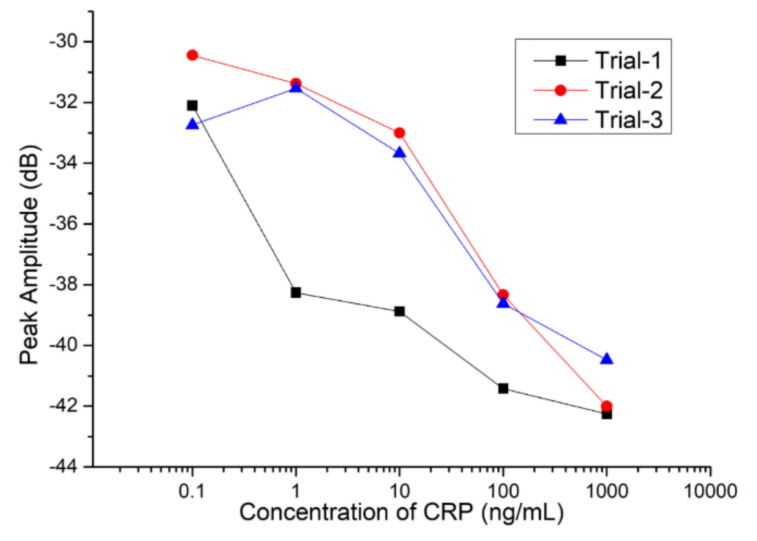
The amplitude response of SAW sensor for measurements taken three times for each CRP concentration.

Figure 7 showed a large variation in the three measurements for each CRP concentration. One possible reason for a large variation is that the surface liquid residue is not completely dried by blowing a small amount of nitrogen gas for 30 s. It is known that it is difficult to control the same treatment for every experiment. It was noted in this study that the amplitude response of SAW sensor was severely attenuated due to the radiation of the acoustic energy into the liquid if the surface liquid residues were not completely removed. Accordingly, a small amount of liquid residue variation may cause considerable attenuation changes. We were unable to perform real-time detection for the proposed SAW sensor. Therefore, we could not estimate the response time for the proposed SAW sensor. Real-time measurement and the estimation of the response time can be made if we change the substrate of 128-degree Y-cut LiNbO_3_ to the 64-degree Y-cut LiNbO_3_, owing to the in-plane polarization of the wave (love wave).

**Figure 8:**
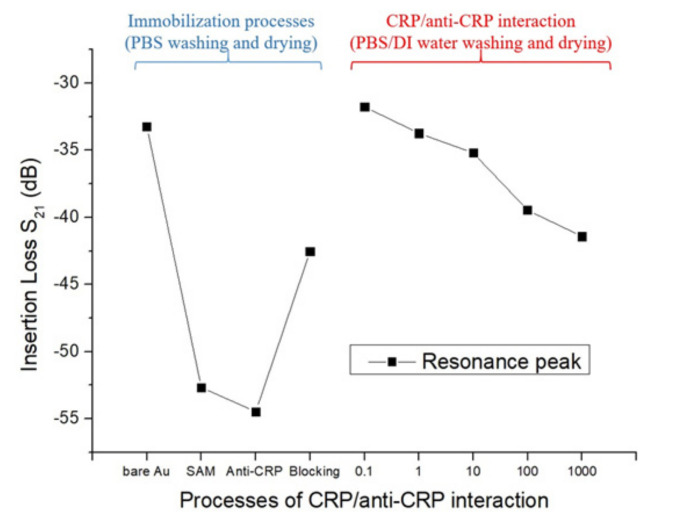
The detailed characterization of the biomolecular membrane modification during the preparation and the subsequent CRP/anti-CRP interactions.

The detailed characterization of the biomolecular membrane modification during the preparation and the following CRP/anti-CRP interactions were also examined, as shown in Figure 8. It is worth noting that wafer-drying had been done before all S-parameter measurements. The insertion loss of −33.24 dB was observed in the bare SAW sensor with an Au-coated layer on top of SiO_2_ guiding layer. It decreased to −52.67 dB after the Au film was immobilized with the 11-MUA containing thiol groups. After activation (EDC/NHS) and the antibody fixation (anti-CRP antibodies) on the self-assembled monolayer (SAM) film, we found that the value of the insertion loss was attenuated to −54.46 dB, as the anti-CRP was linked to the Au surface. A new equilibrium plateau of −42.54 dB was achieved as the SAW sensor was immersed in Ethanolamine-HCl solution and cleaned by PBS. Several factors, such as the improper pH value of the PBS or the residual charge and extension of the polymer brushes, may be responsible for the observation of an apparent increase in the measured insertion loss as the deactivation step was introduced [41,42]. After the immobilization processes, we changed the washing buffer from PBS solution to PBS/DI water, because we found some residues on the sensing area after only PBS washing. To ensure the reliability of the measurement of the CRP/anti-CRP interaction, we added the DI water washing in the cleaning processes, which might affect the mass changes or the pH values of the sensing area. The insertion loss of the SAW sensors with a CRP concentration of 0.1 μg/mL was increased to -31 dB. Further, the insertion loss was continuously attenuated from −31 dB to −41 dB by increasing CRP concentrations for the specific recognition.

The sensitivity of proposed SAW sensor was estimated by partially linear fitting to amplitude attenuation at low concentrations (i.e., 0.1, 1, and 10 μg/mL) [43]. The corresponding slope is taken as a measure for the sensitivity of the commercial standard CRP solution. The sensitivity of proposed SAW sensor for CRP detection is about 0.2711 dB/μg. It is noted that this is just an estimated value. It may have a large error owing to a large variation of amplitude response. Recently, Khan et al. [44] reported that bovine serum albumin (BSA, 1%) was immobilized as an effective enhancer to obtain ultrasensitive detection with high selectivity in comparison with the reported ethanolamine (EA, 1%). To enhance the sensitivity, BSA may be a good choice for blocking the proposed SAW sensor. It is well known that blocking methods have the advantage of washing away unbound and non-specific binding of antibody active sites from the surface. In reference [45], it is mentioned that the use of long chain blocking agents helps to orient the antibody to extend further into the solvent phase. This will make it more favorable for specific hybridization. In addition, a higher hydrophilicity will help the CRP in an aqueous solution to move closer to the electrode surface. It means that the CRP is closer to the immobilized antibody and thus can result in a more efficient hybridization. Therefore, different blocking agents have been used to minimize the non-specific binding onto the sensor surface and to improve the selectivity and sensitivity of the biosensor. To achieve higher sensitivity and lower detection limit, Khan et al. also reported that the optimization of the concentration of antibody and the incubation time of detecting antigens was an important factor and should be considered cautiously. In the future, except the use of BSA for blocking, we can also optimize the concentration of anti-CRP and the incubation time of detecting CRP solution for the proposed SAW sensor.

## 4. Conclusions

In this study, we were able to successfully fabricate a high sensitivity SAW sensor for CRP detection. A good linear relationship between the detected amplitude and the concentrations of CRP from 0.1 μg/mL to 1 mg/mL was observed. This linear regression line is useful to estimate the CRP concentration by using the proposed SAW sensor. The capability of the proposed SAW sensor for detecting a wide range of CRP concentrations covers the clinically relevant range (1–200 μg/mL). The detectable CRP concentration of 0.1 μg/mL in the proposed SAW sensor is high enough for clinical applications. In the future, we plan to use the SAW sensor for a blood test to diagnose the risk of cardiovascular diseases.

## Figures and Tables

**Table 1:** The averaged values of the peak amplitude and the related standard deviations with different CRP concentrations.

CRP Concentration (μg/mL)	Average Peak Amplitude (dB)	Standard Deviation (dB)
0.1	−31.763	1.187
1	−33.726	3.93
10	−35.184	3.213
100	−39.5	1.706
1000	−41.413	0.884
